# Clinical and cost-effectiveness of internal limiting membrane peeling for patients with idiopathic full thickness macular hole. Protocol for a Randomised Controlled Trial: FILMS (Full-thickness macular hole and Internal Limiting Membrane peeling Study)

**DOI:** 10.1186/1745-6215-9-61

**Published:** 2008-11-03

**Authors:** Noemi Lois, Jennifer Burr, John Norrie, Luke Vale, Jonathan Cook, Alison McDonald, the Full-thickness macular hole and Internal Limiting Membrane peeling Study (FILMS) Group

**Affiliations:** 1 Ophthalmology Department, Grampian University Hospitals-NHS Trust, Aberdeen, AB25 2ZN, UK; 2 Health Services Research Unit, University of Aberdeen, Foresterhill, Aberdeen, AB25 2ZD, UK

## Abstract

**Background:**

A full-thickness macular hole (FTMH) is a common retinal condition associated with impaired vision. Randomised controlled trials (RCTs) have demonstrated that surgery, by means of pars plana vitrectomy and post-operative intraocular tamponade with gas, is effective for stage 2, 3 and 4 FTMH. Internal limiting membrane (ILM) peeling has been introduced as an additional surgical manoeuvre to increase the success of the surgery; i.e. increase rates of hole closure and visual improvement. However, little robust evidence exists supporting the superiority of ILM peeling compared with no-peeling techniques. The purpose of FILMS (Full-thickness macular hole and Internal Limiting Membrane peeling Study) is to determine whether ILM peeling improves the visual function, the anatomical closure of FTMH, and the quality of life of patients affected by this disorder, and the cost-effectiveness of the surgery.

**Methods/Design:**

Patients with stage 2–3 idiopathic FTMH of less or equal than 18 months duration (based on symptoms reported by the participant) and with a visual acuity ≤ 20/40 in the study eye will be enrolled in this FILMS from eight sites across the UK and Ireland. Participants will be randomised to receive combined cataract surgery (phacoemulsification and intraocular lens implantation) and pars plana vitrectomy with postoperative intraocular tamponade with gas, with or without ILM peeling. The primary outcome is distance visual acuity at 6 months. Secondary outcomes include distance visual acuity at 3 and 24 months, near visual acuity at 3, 6, and 24 months, contrast sensitivity at 6 months, reading speed at 6 months, anatomical closure of the macular hole at each time point (1, 3, 6, and 24 months), health related quality of life (HRQOL) at six months, costs to the health service and the participant, incremental costs per quality adjusted life year (QALY) and adverse events.

**Discussion:**

FILMS will provide high quality evidence on the role of ILM peeling in FTMH surgery.

**Trial registration:**

This trial is registered with Current Controlled Trials ISRCTN number 33175422 and Clinical Trials.gov identifier NCT00286507.

## Background

An idiopathic full-thickness macular hole (FTMH) represents a defect in the area of maximal vision of the retina, the fovea. If left untreated, FTMH usually leads to severe visual impairment, with over a third of patients experiencing a drop in vision to levels of 20/200 or worse [1]. FTMH are common. The incidence has not been reported in the UK but it is estimated to be around 3/10,000/year based on reported incidence in a similar population in the USA [2]. Macular hole surgery represents one of the most common procedures performed by vitreo-retinal surgeons [3].

Four stages (1–4) of FTMH have been described, often with increasing severity of visual loss as the stage of the hole progresses [4]. Around 40% of cases are likely to progress beyond stage 1 [5,6]. Up to 20% of affected people will develop a FTMH in both eyes [7].

Randomised controlled clinical trials conducted in the 1990s showed that surgery was effective for stage 2, 3 and 4 FTMH [8,9]. Several observational studies have suggested that peeling the internal limiting membrane (ILM) of the retina may improve the visual and anatomical success of the surgery (reviewed by Abdelkader and Lois) [10,11]. However, it is difficult to interpret the results of these studies and compare them with those from other series in which the ILM was not peeled since parameters which are likely to influence anatomical and visual outcomes, including stage of the hole, pre-operative vision, duration of symptoms, size of the macular hole, presence of cataract, and extent of ILM peeling varied among study populations.

Recently, data from two randomised controlled trials (RCT) evaluating the effect of ILM peeling in macular hole surgery have become available [12] (La Cour M, Personal communication, Combined meeting of the Club Jules Gonin and the Retina Society, Cape Town, October 15–20, 2006). Kwok and collaborators reported statistically significantly higher rates of macular hole closure and visual acuity improvement following ILM peeling compared with a no-peeling technique in an RCT involving 49 participants (51 eyes) with stage 2, 3 and 4 FTMH [12]. A high proportion of patients in this RCT had FTMH on stage 4 (50% and 32% in the ILM peel and no ILM peel group, respectively) and few had FTMH on stage 2 (15.4% and 12% in the ILM peel and no ILM-peel groups, respectively). The timing of final outcome assessment was variable, ranging from 6–23 months. La Cour and associates (La Cour M, Personal communication, Combined meeting of the Club Jules Gonin and the Retina Society, Cape Town, October 15–20, 2006) also found a higher rate of macular hole closure following ILM peeling in an interim analysis of an RCT when only 22 patients were enrolled. Based on these results, further recruitment for this RCT has been halted. Another RCT evaluating the effect of ILM peeling in macular hole surgery in patients with large idiopathic macular holes (>400 microns) is currently underway , with a recruitment target of 80 patients.

Peeling the ILM is a surgical technique that can be technically challenging, even for experienced vitreo-retinal surgeons, unless a dye is used to stain the ILM. Indocyanine green has been used for several years to facilitate the visualisation and removal of the ILM during macular hole surgery. However, recent research suggests that Indocyanine green may be toxic to retinal pigment epithelium (RPE) and ganglion cells, and concerns now exist regarding its used for this purpose (revised by Abdelkader and Lois) [10,13]. New dyes have been introduced, including Infracyanine Green [14] Trypan blue [15]. Although long-term data is still needed, these latter dyes seem to be safe and appear to be a good alternative to Indocyanine Green [10].

Some concerns have been raised with regards to the possible damaging effect of ILM peeling in the nerve fibre layer of the retina, which potentially could have adverse effects on central vision [16,17]. There is uncertainty in the literature and among vitreo-retinal surgeons about the balance of potential benefits and adverse effects of ILM peeling in FTMH surgery for stage 2–3 holes. Some surgeons prefer to perform ILM peeling in all cases, whereas others reserve ILM peeling to treat large and/or long-standing macular holes. An Ophthalmology Technology Assessment reviewed the efficacy and safety of macular hole surgery [18] concluded that further research was needed to address whether ILM peeling offered surgical and functional benefit to patients with FTMH.

The hypothesis tested in FILMS is that ILM peeling is superior to the non-peeling procedure with regards to improving vision and quality of life, achieving macular hole closure and that is cost effective.

## Methods and design

### 1. Trial recruitment

Participants will be identified in general and specialised ophthalmic clinics of all eight hospitals participating in this trial: Aberdeen and Dundee (Scotland); Bristol, Sunderland, Oxford, Liverpool, (England); Dublin and Waterford (Ireland).

#### 1.1. Inclusion and exclusion criteria

Eligible participants are those with idiopathic FTMH in stages 2–3, of less or equal than 18 months duration (based on symptoms reported by the patient) and with a visual acuity equal to or worse than 20/40 in the study eye. If bilateral idiopathic FTMH are present, the eye with most recent onset of visual loss will be randomised for the study. Individuals with FTMH in stages 1 and 4, those with idiopathic FTMH stages 2–3 but longer than 18 months duration or with other causes of decreased vision (e.g. corneal scarring, age-related macular degeneration, diabetic retinopathy, glaucoma if central and/or paracentral absolute visual field defects are present) and those with FTMH related to high myopia (> 6 dioptres) or trauma will be excluded from the study. Individuals that do not understand English and those unable to give informed consent will not be included.

#### 1.2. Informing potential participants about the trial

The local clinical investigator (consultant vitreo-retinal surgeon or a doctor/nurse/optometrist working with the consultant) will describe the study face-to-face to potentially eligible participants. Verbal information will be supported by a patient information leaflet containing information about macular holes and current surgical treatments.

#### 1.3. Consent to participate

Once eligibility has been confirmed, the local clinical investigator will ask if the potential participant is interested in joining the trial. If so, the participant will be given a consent form. After checking with the participant that the consent form is understood, the consultant vitreo-retinal surgeon will invite the participant to sign the form and will countersign it. One copy of the consent form will be given to the patient, another will be filed in the hospital case notes, and the third will be kept at the Trial Office (Centre for Healthcare Randomised Trials (CHaRT), Health Services Research Unit, University of Aberdeen, Scotland).

#### 1.4 Information collected at trial entry

Once a participant agrees to join the trial, research optometrists and vitreo-retinal surgeons at each centre will record baseline data on FILMS case report forms (CRF) including full name, address and telephone number, date of birth, gender, hospital number and general practitioner's contact details, and check that eligibility criteria are met prior to enrolment on the study. CRFs are contained in booklets (one booklet per participant recruited), with triplicates of each visit CRF (one to keep in the booklet, one to file in the patient's medical records and one to be sent to the Trial Office) with the exception of the surgical CRF (see below) for which there are only two copies (one for the surgeon and one to be sent by the surgeon to the Trial Office to maintain masking of the participant and optometrist) in each booklet. Some demographic data (gender), the date of attempted recruitment, the centre, and the grounds for non-inclusion will be collected also on ineligible and unwilling participants as well as on participants not recruited for other reasons.

Within 2 weeks (± 2 weeks) prior to the date scheduled for the surgery baseline data on primary and secondary outcomes studied (Table 1) will be collected and recorded on the appropriate CRF for each participant. In addition, information on duration of symptoms related to the macular hole and lens status (phakic, pseudophakic or aphakic) and lens grading in phakic eyes, based on the Age Related Eye Disease Study (AREDS) classification, will be also collected [19].

**Table 1:** Summary of outcome measures, tools used to obtain them, and eye in which they were assessed at baseline and at each follow-up visit

**Outcome measure**	**Tool**	**Eye**	**Baseline**	**1 month**	**3 months**	**6 months**	**24 months**
Distance VA	ETDRS charts	Study eye Contralateral eye	X X		X X	X X	X X

Near VA	Bailey-Lovie charts	Study eye Contralateral eye	X X		X X	X X	X X

Contrast sensitivity	Pelli-Robson chart	Study eye Contralateral eye	X X			X X	

Reading speed	MN Read chart	Study eye Contralateral eye	X X			X X	

Stage of FTMH^•^	Slit-lamp biomicroscopy	Study eye	X				

Status of FTMH	Slit-lamp biomicroscopy^+^ Colour and red free photographs^#^	Study eye Study eye	X X	X X	X X	X X	X X

HRQoL	NEI-VFQ-25 and EQ-5D questionnaires	N/A	X			X	X

Costs:							
- Primary care	Health Service Utilisation questionnaire	N/A				X	
- Secondary care	Targeted questions in CRF	N/A			X	X	X
- Participants	Participant Unit Cost questionnaire	N/A				X	

VA = visual acuity; ETDRS = Early Treatment Diabetic Retinopathy Study visual acuity charts; FTMH = full-thickness macular hole; HRQoL = health related quality of life; N/A = not applicable; ^• ^= Stage 2: absence of posterior vitreous detachment and whenever the largest diameter of the hole is ≤ 400 microns; Stage 3: absence of posterior hyaloid separation but when the largest diameter of the hole is > 400 microns, as classified by the vitreo-retinal surgeon; ^+ ^= closed, open with subretinal fluid around it or open with no subretinal fluid around it as determined by the vitreo-retinal surgeon; ^# ^= size of the macular hole and status of the macular hole (closed, open with subretinal fluid around it or open with no subretinal fluid around it) as determined by a masked observer at the University of Aberdeen.

### 2. Trial interventions

All surgeries will be performed by a consultant vitreo-retinal surgeon or by an experienced vitreo-retinal fellow, supervised by the consultant vitreo-retinal surgeon. All phakic participants will undergo cataract extraction by means of phacoemulsification and intraocular lens implantation at the time of the macular hole surgery.

Participants will be randomised to receive macular hole surgery either with ILM peeling or without ILM peeling. In participants not receiving ILM peeling, a pars plana vitrectomy will be performed, including detachment and removal of the posterior hyaloid, followed by a fluid-air exchange and air-gas (12% C_3_F_8_) exchange. In participants undergoing ILM peeling, a pars plana vitrectomy, including detachment and removal of the posterior hyaloid, will be similarly performed but, in addition, following ILM staining with Trypan blue (Membrane Blue, DORC) the ILM will be peeled off the retina, in an area of around 1–2 disc diameters around the hole. If staining of the ILM is considered inadequate, re-staining is permitted. The surgery will be completed by a fluid-air exchange and an air-gas (12% C_3_F_8_) exchange. Details of the surgery will be recorded in the appropriate case report form. To assure masking of the optometrists and participants (see below) to the allocated treatment, once the CRF for the surgery is filled one copy will be removed from the CRF booklet and kept locked by the vitreo-retinal surgeon and the other will be sent immediately to the Trials Office.

The surgical CRF contains information on date of admission; grade of the surgeon, assistant surgeon and anaesthetist [consultant, staff grade or associate specialist (non-training posts) or registrar or senior house officer (training posts)]; type of anaesthesia; performance or lack of performance of ILM peeling around the hole and the degree of completeness of this manoeuvre (peel complete or incomplete); confirmation that the vitreous was attached at the time of the surgery and any complications occurring intraoperatively. In addition, the time at which the participant enters the anaesthetic room, the "operating time", defined as the time between starting the case (getting the operating microscope over the participants' eye, ready for the surgery) and completing the case (moving the microscope away from the participant's eye at the end of the procedure), and the time at which the participant leaves the theatre (or the recovery room when appropriate) will be recorded. The date of discharge from the hospital will also be recorded.

All participants will be instructed to posture face down as soon as possible after the surgery for 45 minutes every hour for a period of 5–7 days. Each participant will be given a posturing chart to record the time postured during these 5–7 days, which will be sent, once filled, to the central Trials office in Aberdeen. In addition, at the 1 month follow-up visit the participant will be asked how many days in total did they posture following surgery (in case they have not completed and returned the posturing chart).

### 3. Treatment allocation

The existing central randomisation service (fully automated telephone randomisation) in the Centre for Healthcare Randomised Trials (CHaRT), Health Services Research Unit at the University of Aberdeen, will be used to randomise participants. A minimisation algorithm (according to Taves, with p = 1)^20^, considering trial centre, distance visual acuity in the study eye and in the fellow eye (20/40–20/160; 20/200–20/500; <20/500), duration of the macular hole (= 1 year; > 1 year), lens status (phakic;aphakic;pseudophakic) and stage of the hole (Grade 2; Grade 3), as classified by the vitreo-retinal surgeon will be used.

### 4. Masking of intervention and outcome assessment

Patients, optometrists obtaining data on visual function, photographers and the observer at the University of Aberdeen responsible for analysing the size and status of the hole will be masked with regards to the type of procedure performed to the participant and will remain masked throughout the study. Only the vitreo-retinal surgeon, for obvious reason, will not be masked to the type of surgery performed.

At enrolment potential participants will be informed that they will be unaware as to whether they had received internal limiting membrane peeling at the time of the macular hole surgery. The surgeons will know which type of procedure they have performed but they will not reveal this to participants. The only scenario in which the participant will be unmasked is if the macular hole is not closed following surgery. In this situation, the participant will be offered further surgery and the nature of this surgery will be discussed with the participant.

### 5. Subsequent arrangements

#### 5. 1. Informing the general practitioner

Following formal trial entry, the sites will contact the general practitioner through the post to let him/her know about the participant's involvement in the trial. The letter sent to the general practitioner includes a brief description of the trial together with a request to notify the Trial Office if any events that may lead to their patient failing to attend any of the follow-up visits.

#### 5. 2. Follow-up

Participants will be followed at 1, 3, 6 and 24 months post surgery. Visits will take place within 2 weeks from the scheduled date. All data will be recorded at each visit in the appropriate CRF. Table 1 summarises the data collected at each of the follow-up visits. Post-operative complications will also be recorded at each visit.

### 6. Data processing

Data from the various sources outlined above will be sent to the Trial Office in Aberdeen for processing. A random 10% sample of that data will be double checked for accuracy. Staff in Aberdeen will work closely with investigators at all participating sites to ensure complete and accurate data recording. Extensive range checks will be conducted to further enhance the quality of the data.

### 7. Outcome assessment

The primary outcome of the study is the mean difference between treatment groups in the Early Treatment Diabetic Retinopathy Study (ETDRS) distance visual acuity score at 6 months.

Secondary outcomes include ETDRS distance visual acuity at 3 and 24 months, near visual acuity at 3, 6 and 24 months, contrast sensitivity at 6 months, reading speed at 6 months, anatomical closure of the macular hole at each time point (1, 3, 6 and 24 months), health related quality of life (HRQOL) at 6 months, costs to the health service and the participant, incremental costs per QALY and adverse events.

A summary of the outcome measures studied and the tools used for their assessment at baseline and at each follow-up visit is provided in Table 1.

### 8. Analysis of the data

#### 8.1. Statistical analysis

The principal analysis will take place when six month data collection is complete. A subsequent analysis will be conducted once 24 months data is available. The statistical analysis will be based on all patients as randomised, irrespective of subsequent treatment received and, thus, will follow the intention to treat principle. Additionally, the primary analysis will be based upon available case data with no imputation of missing values. Sensitivity analyses, which impute extreme values will be performed for the primary outcome (visual acuity at 6 months) and for other visual function and quality of life secondary outcomes as appropriate. Statistical significance for the primary and secondary outcomes will be based on two-sided tests with 2P ≤ 0.05 taken as the criterion for statistical significance. The primary outcome of ETDRS visual acuity score at 6 months will be compared between the two groups (ILM peel and no ILM peel) using a generalised linear model to calculate the mean difference adjusted for the baseline score and minimisation covariates. The primary outcome will additionally be analysed according to the pre-specified subgroups (see Treatment Allocation section, minimisation criteria, above) by including corresponding interaction term(s) in the regression model. For these subgroup analyses, stricter criteria for statistical significance (2P ≤ 0.01) will be applied. Secondary outcomes, adverse events and re-operation rates will be analysed using generalised linear model with adjustment for minimisation covariates as appropriate. Analyses of health related quality of life measures (EQ-5D and VFQ-25) and secondary measures of visual function (contrast sensitivity, MN Reading speed and distance and near visual acuity) will be adjusted for the corresponding baseline score in addition to the minimisation variables.

#### 8.4 Economic analysis

##### 8.4.1 Estimation of costs

Costs that fall on both the participant and the health service will be elicited. Participant costs will comprise three main elements: self purchased healthcare; travel costs for making return visit(s) to NHS health care; and time costs of travelling and attending NHS health care.

Self-purchased health care will include items such as prescription costs and over the counter medications. Information about these will be collected in the Participant Unit Cost questionnaire, which will be sent by post to each participant prior to the 6-month visit, and which will be returned, once filled by the participant, to the central office in Aberdeen. Estimation of travel costs requires information from participants about the number of visits to their general practitioner, ophthalmologist or optometrists and the unit cost of making a return journey to each type of health care provider. The cost of participant time will be estimated in a similar manner. The participant will be asked, in the Participant Unit Cost Questionnaire, how long they spent travelling to and attending their last visit to each type of health care provider. Participants will be asked also what activity they would have been undertaking (e.g. paid work, leisure, housework) had they not attended the health care provider. These data will be presented in their natural units, e.g. hours, and also costed using standard economic conventions, e.g. the Department of Transport estimates for the value of leisure time. These unit time costs, measured in terms of their natural and monetary terms, will then be combined with estimates of number of health care contacts derived from the health care utilisation questions.

Health service costs of surgery will be recorded prospectively for every participant in the study. Main areas of costs are operation costs include staff, consumables, capital and overheads.

Use of secondary care services following the operation will be collected on the 3, 6 and 24 month CRFs. Information on non-protocol (protocol visits are those scheduled for the purposes of data collection) eye-related visit(s) to the outpatients/casualty department or hospital admission(s) and need for further surgery(ies) will be also collected.

Use of primary care services such as prescription medications, contacts with primary care practitioners e.g. general practitioners, practice nurses, optometrists and ophthalmologists will be collected via the Health Care Unit Cost questionnaire administered at 6 months follow-up.

##### 8.4.2 Estimation of effectiveness

In the economic analysis effectiveness at 6 and 24 months will be measured in terms of quality adjusted life years (QALYs). QALYs will be estimated by the participants' responses to the EQ-5D questionnaire (administered at baseline, 6 and 24 months). The estimation of QALYs will take account of the mortality of study participants. Participants who die within the study follow-up will be assigned a zero utility weight from their death until the end of the study follow up. QALYs before death will be estimated using linear extrapolation between the QALY scores at baseline and all available EQ-5D scores up to death or the end of the study follow-up. The method of eliciting QALYs described is one commonly adopted in economic evaluation [21].

##### 8.4.3 Estimation of cost-effectiveness

The primary analysis will be based on six-month follow-up of the trial for the incremental cost per QALY. A second analysis will be conducted for a 24-month time horizon using the same methods as outlined in this manuscript. The results will be presented as point estimates of mean incremental costs, QALYs, and incremental cost per QALY. Measures of variance for these outcomes are likely to involve bootstrapping estimates of costs, QALYs, and incremental cost per QALY. Incremental cost-effectiveness data will be presented in terms of cost-effectiveness acceptability curves (CEACs).

Other forms of uncertainty, e.g. concerning the unit cost of a resource from the different centres, will be addressed using standard deterministic sensitivity analysis. Where feasible the results of the sensitivity analyses will also be presented as CEACs.

A secondary economic analysis will be presented. In this analysis the costs and consequences measured in terms of visual function and adverse events will be presented as a cost-consequence analysis. Where appropriate uncertainty will be explored using similar approaches to those outlines above.

### 9. Sample size

It was estimated that sixty-four participants will be required in each group, assuming a common standard deviation of 12 ETDRS points in these two randomised groups, to detect a 6 ETDRS score difference (an effect size of 0.5) using a two sample two sided t-test at a 5% level of significance and 80% power (128 patients). This calculation was based on estimated mean post treatment EDTRS visual acuity scores and the corresponding SDs for each intervention arm from published studies of alternative interventions for macular hole surgery [22,23]. To allow for a loss to follow up of 10% we aim to recruit a total of 140 participants into the trial.

For the subgroup analysis of stage, if 70% of the participants are recruited with the more severe stage 3 macular holes, the study will have 80% power to detect an effect size of 0.6. If a common standard deviation of 12 points is assumed, there will be sufficient power to detect a mean difference of 7 EDTRS letters between the randomised groups.

### 10. Organisation

#### 10.1 Local organisation

Each collaborating centre will identify a ***clinical co-ordinator***. The responsibilities of this person will be to 1) establish the trial locally, 2) take responsibility for clinical aspects of the trial locally (for example, if any particular concerns emerge), 3) notify the Trial Office of any suspected unexpected serious adverse event which might be related to trial participation, 4) ensure masking of the optometrist and participant to the allocated surgery.

Each participating centre will appoint a ***study optometrist ***to co-ordinate the day-to-day aspects of the trial. The responsibilities of this person are 1) to conduct visual assessment of the participants involved in the trial, 2) telephone the Trial Office to obtain randomisation and allocate treatment for participants enrolled in the study, and to let the vitreo-retinal surgeon know about it before the surgery, 3) arrange follow-up appointments, 4) keep regular contact with the local clinical co-ordinator, with notification of any problem or unexpected development, 5) maintain regular contact with the Trial Office, 6) provide information about the trial to the participants, 7) ensure CRFs are completed and sent to the Trial Office, 8) ensure that enrolled participants will undergo macular hole surgery within 2 weeks from the baseline assessment, 9) be masked to the allocated treatment, and 10) clarify the situation when the Trial Office fails to make a contact with a local participant, getting in touch by phone or a visit, if necessary.

#### 10. 2 Trial co-ordination

##### 10. 2. 1. The Trial Office

The Trial Office at the Centre for Healthcare Randomised Trials (CHaRT), at the Health Services Research Unit, University of Aberdeen, will provide the day-to-day support to the clinical centres. CHaRT will be responsible for collection of data (in collaboration with the local study optometrists), data processing and analysis. It will be responsible for randomisation, distribution and collation of CRFs and questionnaires.

##### 10. 2. 2. The Project Management Group

The day to day running of the trial will be co-ordinated by its Project Management Group. This consists of the chief investigator Noemi Lois, together with Jennifer Burr, John Norrie, Luke Vale, Jonathan Cook, Alison McDonald, Gladys McPherson, Charles Boachie and Laura Ternent.

#### 10.3 Data and safety monitoring

##### 10. 3. 1. The Data Monitoring Committee

The data monitoring committee (DMC) will comprise of a statistician, Prof. Gordon Murray (chair) and by two vitreo-retinal surgeons, Mr William Aylward and Mr Tom Williamson.

##### 10. 3. 2. Other safety concerns

Collaborators and participants can contact the trial office or the chief investigator about any worries they may have about the trial.

FILMS will be conducted according to the MRC Good Clinical Practice Guidelines (1998) and the Data Protection Act (1998). Approval of The Multicentre Research Ethics Committee, the Local Ethics Committees and local hospital trusts of each participating centre has been obtained.

### Publication

The success of the trial depends entirely on the collaboration of a large number of participants, and a group of Vitreo-Retinal surgeons and optometrists. For this reason, chief credit for the trial will be given, not to the committees or central organisers, but to all those who have collaborated in the trial (the FILMS Study Group). The trial's publication policy is described in detail in the Site File and follows the rules of the International Committee of Medical Journal Editors. The results of the trial will be reported first to trial collaborators. The main report will be drafted by the Project Management Group, and circulated to all clinical co-ordinators and members of the DMC for comments. The final version will be agreed by all before submission for publication, on behalf of the collaboration.

To safeguard the integrity of the main trial, reports of explanatory or satellite studies would not be submitted for publication without prior discussion with the Project Management Group.

Once the main report has been published, a summary will be sent to the GP of participants involved in the trial and to individual participants' or relatives' who had indicated they would wish to receive one.

## Abbreviations Used

FTMH: Full-thickness macular hole; RCT: Randomised controlled trials; ILM: Internal limiting membrane; FILMS: Full-thickness macular hole and Internal Limiting Membrane peeling Study; UK: United Kingdom; HRQOL: Health related quality of life; QALY: Quality adjusted life year; RPE: Retinal pigment epithelium; CHaRT: Centre for Healthcare Randomised Trials; CRF: Case report forms; AREDS: Age-related eye disease study; CEACs: Cost-effectiveness acceptability curves.

## Competing interests

The authors declare that they have no competing interests.

## Authors' contributions

NL conceived the study, participated in its design and coordination and drafted this manuscript. JB participated in the design and coordination of the study and contributed to this manuscript. JN and LV participated in the design of the study and contributed to the manuscript. JC participated in the design and statistical analysis plan and contributed to the manuscript. AM participated in the design and coordination of the study and contributed to the manuscript. All authors read and approved the final manuscript.

For the Full-thickness macular hole and Internal Limiting Membrane peeling Study (FILMS) Group

Aberdeen – Hatem Atta, Charles Boachie, John Forrester, Karon McEwing, Laura Duncan, Ayyakkawnv Manivannan, Gladys McPherson, Laura Ternent, Alison Farrow; Bristol – Andrew Dick, Richard Haynes, Cherry Daly, Gillian Bennerson, Stephen Neilson; Dublin – Dara Kilmartin, Catherine Cleary, Tarik Saddik; Dundee – John Ellis, Stan Keys, Jim Talbot; Liverpool – Ian Pearce, Carl Groenewald, David Wong, Henrich Heimann, Valerie Tompkin, Ronnie Jackson; Oxford – CK Patel, Charles Cottriall, Lynda Lindsell, Anne Bolton; Sunderland – David Steel, Sarah Muir, Anita Murphy, Terri Ainsley; Waterford – Stephen Beatty, Asif Orakzai, Ayman Saeed, Muhammad Irfan Khan
